# Analysis of the evolution of public policies and strategies for eliminating vertical transmission of the human immunodeficiency virus: a narrative review, Brazil, 1985-2025

**DOI:** 10.1590/S2237-96222026v35e20250576.en

**Published:** 2026-07-20

**Authors:** Andréa Mônica Brandão Beber, Katia Cristina Bassichetto, Francisca Lidiane Sampaio Freitas, Ximena Pamela Díaz Bermúdez, Elisangela da Silva, Lino Neves da Silveira, Leila Posenato Garcia, Adele Schwartz Benzaken

**Affiliations:** 1 Universidade de Brasília Faculdade de Ciências da Saúde Brasília DF Brazil Universidade de Brasília, Faculdade de Ciências da Saúde, Brasília, DF, Brazil; 2 Santa Casa de Misericórdia de São Paulo Faculdade de Ciências Médicas São Paulo SP Brazil Santa Casa de Misericórdia de São Paulo, Faculdade de Ciências Médicas, São Paulo, SP, Brazil; 3 Universidade de Brasília Programa de Pós-Graduação em Saúde Coletiva Brasília DF Brazil Universidade de Brasília, Programa de Pós-Graduação em Saúde Coletiva, Brasília, DF, Brazil; 4 Universidade do Estado do Amazonas Programa de Pós-Graduação em Medicina Tropical Manaus AM Brazil Universidade do Estado do Amazonas, Programa de Pós-Graduação em Medicina Tropical, Manaus, AM, Brazil; 5 Secretaria de Estado da Saúde do Distrito Federal Brasília Brasília DF Brazil Secretaria de Estado da Saúde do Distrito Federal, Brasília, DF, Brazil; 6 Fundação Jorge Duprat Figueiredo de Segurança e Medicina do Trabalho Escritório Avançado em Santa Catarina Florianópolis SC Brazil Fundação Jorge Duprat Figueiredo de Segurança e Medicina do Trabalho, Escritório Avançado em Santa Catarina, Florianópolis, SC, Brazil; 7 AIDS Healthcare Foundation Los Angeles Los Angeles CA United States AIDS Healthcare Foundation, Los Angeles, CA, United States

**Keywords:** Health Policy, Infectious Disease Transmission, Vertical, HIV, Maternal and Child Health, Narrative Review, Política de Salud, Transmisión Vertical de Enfermedad Infecciosa, VIH, Salud Materno-Infantil, Revisión Narrativa

## Abstract

**Objective:**

To analyze the evolution of Brazilian public health policies and strategies aimed at eliminating vertical transmission of the human immunodeficiency virus (HIV), the contribution of these policies to the reduction of AIDS cases among children under 5 years old, and the challenges to achieving the global elimination goals by 2030.

**Methods:**

This is a narrative review of publications from 1985 to 2025, based on consultation of official Ministry of Health documents and scientific articles.

**Results:**

Analysis of 45 publications revealed Brazil’s progress in addressing vertical transmission of HIV, with the free provision of supplies for HIV prevention, diagnosis and treatment in the Brazilian Unified Health System. Implementation of strategic projects, such as Nascer-Maternidades and Rede Cegonha, and the subnational certification process in priority municipalities contributed to the achievement of the country’s global goals for eliminating vertical transmission of HIV in 2025. Social inequities such as race/skin color and social class, regional disparities in prenatal care, weaknesses in surveillance systems and barriers to access to health services remain challenges to the equitable elimination of this disease.

**Conclusion:**

The public policies adopted for maternal and child health were fundamental to Brazil’s achievement of international certification for the elimination of vertical HIV transmission as a public health problem. Intensifying mobilization and continuing education programs, strengthening local management, and ensuring equity of care are necessary to achieve elimination targets nationwide.

## Resumen

**Objetivo:**

Analizar la evolución de las políticas y estrategias de salud pública brasileñas dirigidas a la eliminación de la transmisión vertical del virus de la inmunodeficiencia humana (VIH), su contribución a la reducción de casos de sida en niños menores de 5 años y los desafíos para alcanzar las metas globales de eliminación para 2030.

**Métodos:**

Revisión narrativa de publicaciones de 1985 a 2025, basada en la consulta de documentos oficiales del Ministerio de Salud y artículos científicos.

**Resultados:**

El análisis de 45 publicaciones reveló el progreso de Brasil en la respuesta a la transmisión vertical del VIH, con la provisión gratuita de insumos para la prevención, el diagnóstico y el tratamiento del VIH en el Sistema Único de Salud. La implementación de proyectos estratégicos, como Nascer-Maternidades y Rede Cegonha, y el proceso de certificación subnacional en municipios prioritarios contribuyeron al logro de las metas globales del país para la eliminación de la transmisión vertical del VIH en 2025. Las desigualdades sociales, como la raza/color de piel y la clase social, las disparidades regionales en la atención prenatal, las deficiencias en los sistemas de vigilancia y las barreras de acceso a los servicios de salud, siguen siendo obstáculos para la eliminación equitativa de esta enfermedad.

**Conclusión:**

Las políticas públicas adoptadas en el área de salud maternoinfantil fueron fundamentales para que Brasil alcanzara la certificación internacional de eliminación de la transmisión vertical del, como problema de salud pública. Intensificar la movilización y los programas de educación continua, fortalecer la gestión local y garantizar la equidad en la atención son necesarios para alcanzar las metas en todo el territorio nacional.

**Ethical aspects**

This research used publicly available and anonymized databases.

## Introduction

Global policies to eliminate vertical transmission of the human immunodeficiency virus (HIV) aim to end new pediatric infections by 2030 (1,2). In recent decades, the World Health Organization (WHO), the Pan American Health Organization (PAHO) and the United Nations Children’s Fund have established standardized criteria for validating the elimination of vertically transmitted diseases as public health problems (3).

This process began in 2005 with the PAHO regional strategic plan for the control of HIV and other sexually transmitted infections in Central America, expanded in 2007 to the Americas, Asia, the Pacific and Africa. Successive editions of the “Global Validation Guide for the Elimination of HIV, Syphilis and Hepatitis B” consolidated international commitment to eliminate these diseases by 2030 (3,4). Aligned with the Sustainable Development Goals, these initiatives resulted in a 62% global reduction in pediatric HIV infections, from 300,000 to 120,000 between 2010 and 2023 (5). By 2024, 19 countries had achieved one of the certification targets, according to different validation criteria (Supplementary Table 1) (6,7).

**Table 1:** Summary description of the bibliographic searches using the Standards for Reporting Literature Searches (Starlite)

S	**Selective sampling strategy** Selection of Ministry of Health technical documents, such as manuals, protocols, ordinances and technical notes containing policies and strategies aimed at reducing and eliminating vertical transmission of the human immunodeficiency virus (HIV). Epidemiological bulletins with the number of AIDS cases in children under 5 years old. Scientific articles on prenatal services, prevention of vertical transmission of HIV and treatment of pregnant women and children living with HIV.
T	**Types of publications** Descriptive and evaluative studies, literature reviews, as well as manuals, protocols, ordinances, technical notes, legislation and epidemiological bulletins.
A	**Approaches** Searches in electronic databases and Ministry of Health websites.
R	**Range of years** 1985-2024: includes data on the number of pediatric AIDS cases, with effect from the first cases recorded in Brazil. 1995-2024: covers the period from the publication of the first national document on programmatic policies and strategies for preventing mother-to-child transmission of HIV up until 2024. 2019-2025: covers Brazilian scientific articles on prenatal care coverage, HIV testing in pregnant women, access to antiretroviral therapy during pregnancy and information on preventing vertical transmission of HIV.
L	**Limits** Brazilian publications, with no language restrictions.
I	**Inclusion and exclusions** Inclusion: technical documents on Brazilian policies for preventing vertical transmission of HIV and epidemiological data on AIDS in children under 5 years old. Exclusion: articles that did not address the topic or did not contain relevant information about gaps in vertical transmission prevention.
T	**Terms used** Official technical documents and data on pediatric AIDS: titles and year of publication. Scientific articles: Health Science Descriptors (Descritores em Ciências da Saúde)/Medical Subject Headings (DeCS/MeSH) and Boolean operators: (((Infectious Disease Transmission, Vertical) OR (Mother-to-Child Transmission OR Vertical Transmission)) AND (HIV)) AND (Brazil). (health coverage) AND (prenatal) AND (Brazil) AND (HIV)
E	**Electronic sources ** Official Ministry of Health technical documents: Virtual Health Library (Biblioteca Virtual em Saúde) and the Ministry of Health website (content repositories, epidemiological surveillance and information systems), including webpages of the former Department of Chronic Diseases and Sexually Transmitted Infections and the current Department of HIV, AIDS, Tuberculosis, Viral Hepatitis, and Sexually Transmitted Infections. AIDS cases data on children under 5 years old: epidemiological bulletins available on the Ministry of Health website, including the same webpages already listed. Peer-reviewed scientific articles held in the following databases: PubMed, Scientific Electronic Library Online and Biblioteca Virtual em Saúde/Literatura Latino-Americana e do Caribe em Ciências da Saúde

In Brazil, since 1990, public health policies have aimed to reduce cases of pediatric AIDS. In 2017, the country adopted strategies to achieve the international goal of eliminating vertical transmission of HIV, aiming to reduce this rate to less than 2% and achieve prenatal care coverage, HIV testing and use of antiretroviral therapy greater than or equal to 95%, for two consecutive years (8). Between 2012 and 2023, the rate of AIDS detection in children under 5 years old decreased by approximately 56%, from 3.4 cases to 1.2 cases per 100,000 inhabitants (9).

Despite these advances, socio-regional barriers persist in equitable access to early diagnosis and treatment during pregnancy. In 2023, the Southeast region of Brazil recorded 31.4% of cases of pregnant women living with HIV, with a detection rate of 5.3 cases per 1,000 live births, a value higher than the national average (3.3/1,000 live births). In the same year, the highest rates of AIDS in children under 5 years old were concentrated in the North and Northeast regions, notably in the states of Roraima (8.2/100,000 inhabitants), Pará (2.9/100,000 inhabitants) and Sergipe (2.4/100,000 inhabitants) (9).

Previous research has analyzed the impact of public policies on HIV indicators in Brazil (10,11), but there is a scarcity of analyses that organize, in a historical and chronological way, the national response to vertical transmission of HIV and its contribution to the reduction of AIDS cases in children. Understanding this trajectory can strengthen adherence to agreed targets, improve regional strategies, and support managers and policymakers in identifying gaps in contexts of inequality, as well as disseminating good practices to other countries and serving as a reference for those seeking to eliminate vertically transmitted diseases.

This study aimed to analyze the evolution of Brazilian public health policies and strategies focused on eliminating vertical transmission of HIV, the contribution of these policies to reducing AIDS cases in children under 5 years old, as well as the challenges to achieving global elimination targets by 2030.

## Methods

### Design

This is a narrative literature review (12,13) developed based on the analysis of technical documents related to Brazilian public policies aimed at preventing vertical transmission of HIV, description of the evolution of AIDS cases in children under 5 years old and identification of Brazilian scientific articles on the subject.

### Search strategy

The search strategy followed the Standards for Reporting Literature Searches (Starlite) (Table 1) (14).

### Collection and selection process 

Two sets of publications were included: (i) manuals, protocols, ordinances and technical notes, published between 1995 – the year the first national guide on prevention of vertical transmission of HIV was launched – and 2024, in addition to epidemiological bulletins with data with effect from the first reports of AIDS cases in children under 5 years old in Brazil, from 1985 to 2024; and (ii) Brazilian studies on coverage of prenatal care, HIV testing among pregnant women, access to antiretroviral therapy during pregnancy and prophylaxis during childbirth and for the newborn.

The technical documents were identified in the Biblioteca *Virtual em Saúde* (Virtual Health Library) and on the Ministry of Health website. Of the 35 official documents selected, 26 were relevant for analyzing policies and strategies for eliminating vertical transmission of HIV.

The search for scientific articles was conducted in PubMed, the Scientific Electronic Library Online, and the *Biblioteca Virtual em Saúde/Literatura Latino-Americana e do Caribe em Ciências da Saúde* (Virtual Health Library/Latin American and Caribbean Literature on Health Sciences), encompassing publications from 2019 to 2025, using the following descriptors: Infectious Disease Transmission, Mother-to-Child Transmission, Vertical Transmission, HIV, Health Coverage, Prenatal, and Brazil. Of the 149 articles found, 59 were excluded for being duplicates or out of scope, 55 after title and abstract analysis, and 16 after full-text review.. Nineteen articles were selected.

In order to organize the two sets of publications mentioned, it was necessary to create intermediate spreadsheets with the following content:

- For technical documents: year, policy/strategy and changes in national guidelines.
- For scientific articles: author, year, coverage, sociodemographic characteristics, prenatal care coverage, HIV testing and treatment, and case management at child delivery.

## Results

The final selection totaled 45 publications, comprising 26 technical documents and 19 scientific articles (Figure 1).

**Figure 1:**
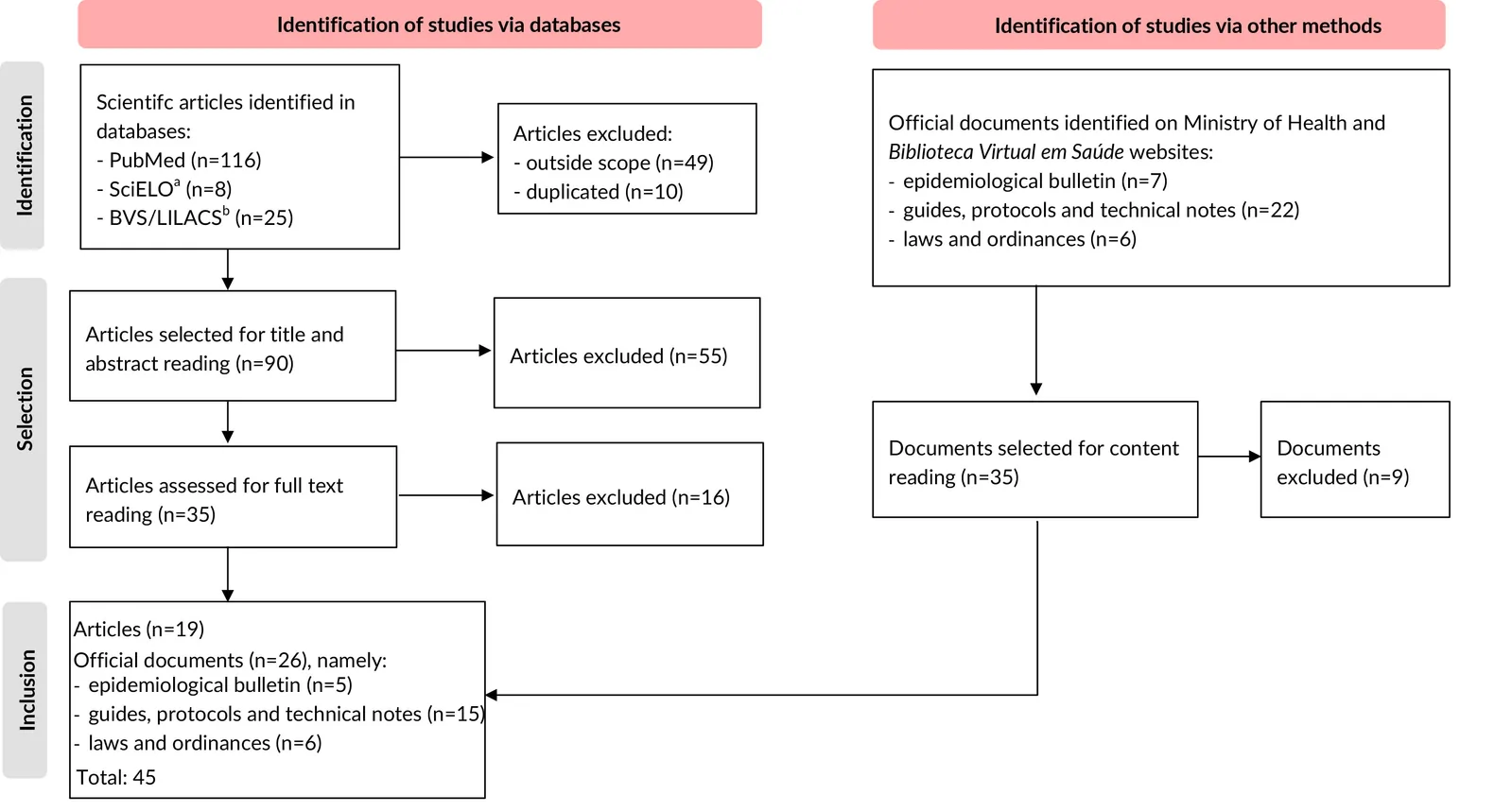
Publication selection process for narrative review of Brazilian public policies to eliminate vertical transmission of the human immunodeficiency virus (HIV). Brazil, 2025

^a^SciELO: Scientific Electronic Library Online; ^b^BVS/LILACS: *Biblioteca Virtual em Saúde/Literatura Latino-Americana e do Caribe em Ciências da Saúde*.

The technical documents were ordered chronologically and categorized according to the epidemiological evolution of AIDS cases in children under 5 years old, concurrently with the main programmatic milestones of the prevention policies and strategies adopted in Brazil (Figure 2). The scientific articles addressed the challenges to achieving the global elimination goals.

**Figure 2:**
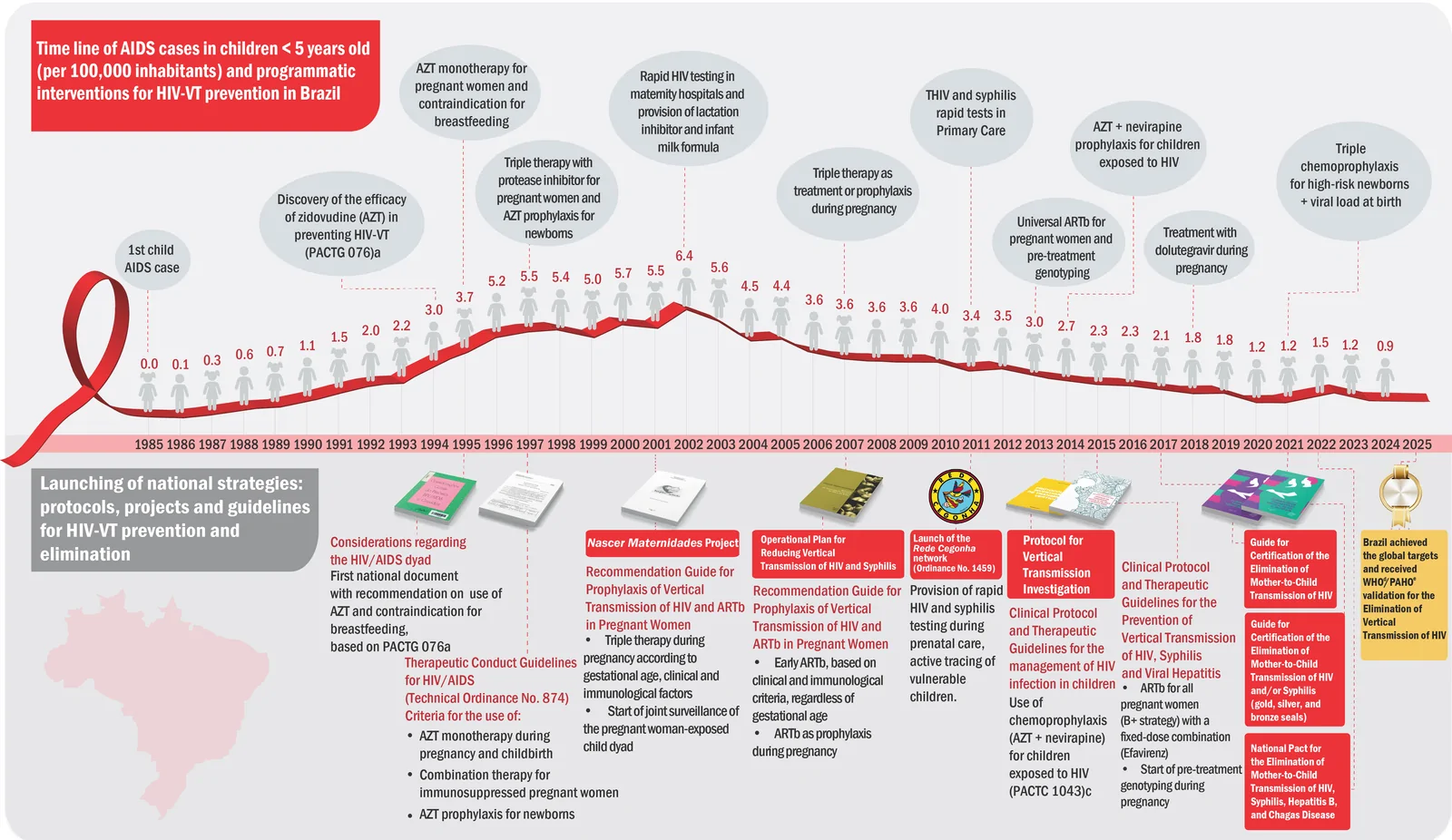
Evolution of the AIDS detection rate in children under 5 years of age (per 100,000 inhabitants), programmatic interventions and national strategies to eliminate vertical transmission of the human immunodeficiency virus (HIV-VT). Brazil, 1985-2025

^a^PACTG 076: Pediatric AIDS Clinical Trials Group Protocol 076; ^b^ART: antiretroviral therapy; ^c^PACTG 1043: Pediatric AIDS Clinical Trials Group Protocol 10431043; ^d^WHO: World Health Organization; ePAHO: Pan American Health Organization.

The review results illustrate the history of the epidemic and show that pediatric AIDS in Brazil was first recorded in 1985, with three cases reported in the state of São Paulo (15). In the following years, the rate in children under 5 years old increased progressively, reaching 3.7 cases per 100,000 inhabitants in 1995, following the expansion of the epidemic among women (16).

Between 1996 and 2002, treatment for AIDS was made available via the Brazilian Unified Health System (*Sistema Único de Saúde*, SUS), but challenges remained such as limited access to HIV diagnosis, low prenatal testing coverage and insufficient adherence to the prophylaxis available at the time (zidovudine/AZT) during childbirth (16). At the end of this period, the pediatric AIDS incidence rate reached its peak in the time series, with 6.4 cases per 100,000 inhabitants, corresponding to 2.9% of people living with HIV in Brazil (17).

From 2003 onwards, a progressive reduction in the rate of AIDS detection in children under 5 years old was observed, falling from 5.6 to 3.4 per 100,000 inhabitants in 2011. This result was possibly associated with the introduction of rapid testing in prenatal care and maternity wards, use of prophylactic antiretroviral therapy during pregnancy, continuing education aimed at health professionals and expansion of knowledge about the disease among the population (18).

From 2012 to 2023, specific actions implemented, such as the universal provision of antiretroviral therapy, early diagnosis and maternal and child prophylaxis stratified by risk, contributed to the reduction in the rate of AIDS detection in children under 5 years old. The rate fell from 3.4 cases to 1.2 cases per 100,000 inhabitants, which enabled the WHO/PAHO impact target to be reached, a scenario that was maintained in 2025 (19).

In all periods analyzed, in order to support decision-making by health service managers and health professionals, the Ministry of Health developed complementary surveillance and monitoring tools for HIV infection cases and AIDS-related deaths in pregnant women and children, accessible by states, municipalities and the Federal District (Figure 3).

**Figure 3:**
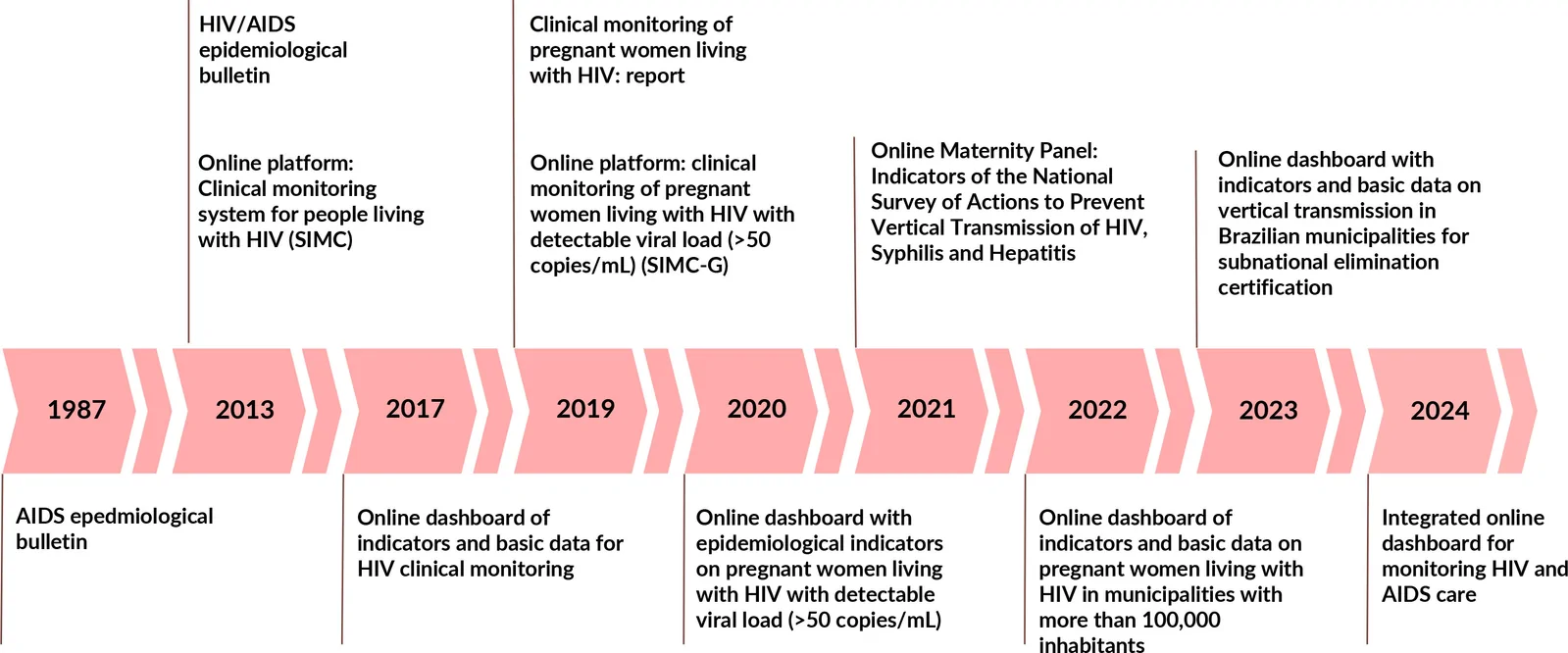
Tools for monitoring pregnant women living with human immunodeficiency virus (HIV), exposed and infected children, according to year of first publication. Brazil, 2025

Strategies for reducing and eliminating vertical transmission of HIV began after the publication of the AIDS Clinical Trial Group Protocol 076 (“PACTG 076”) in 1994 in the United States. This study represented an initial milestone in the case management of pregnant women living with HIV, demonstrating the effectiveness of AZT in reducing vertical transmission of HIV when administered during pregnancy, at delivery, and to non-breastfed newborns (20). The following year, Brazil published its first national guidelines for health professionals with guidelines for the prevention of vertical transmission of HIV, compiled in the document entitled “General considerations on the dyad: HIV/AIDS and pregnancy” (*Considerações gerais do binômio: HIV/aids e gravidez*) (15).

However, the measures were implemented only after the enactment of Law No. 9313/1996, which established the free distribution of antiretrovirals in the SUS and sought to ensure universal access to antiretroviral therapy for people living with HIV with indication for treatment (21). There were, however, uncertainties about the mechanisms of HIV transmission to the baby and the long-term effects of AZT use during pregnancy (22).

Between 1996 and 2001, internationally recognized studies highlighted the importance of maternal virological and immunological factors in vertical transmission of HIV, especially in the final weeks of gestation, during childbirth and during breastfeeding (20,23,24). In 1996, Brazil began offering intravenous AZT in the public health system for prophylaxis during childbirth (25). In 1997, Ministry of Health Ordinance No. 874 reinforced the use of AZT from the 14th week of gestation regardless of clinical and immunological factors, incorporated combination therapy for immunosuppressed pregnant women living with HIV, introduced protease inhibitors for pregnant women who had experienced treatment failure, and extended AZT prophylaxis to newborns from the fourth to the sixth week of life, maintaining the indication for elective cesarean section and contraindication of breastfeeding (26).

In the early 2000s, the increase in HIV cases among women of reproductive age and pregnant women highlighted the need for more robust prevention policies. In response, the Ministry of Health instituted mandatory reporting of pregnant women living with HIV and children exposed to the virus, by means of Ordinance No. 993/2000, which intensified surveillance of the disease and improved care directed at these populations (27).

Although Brazil offered free-of-charge supplies for AIDS diagnosis and treatment in the SUS, pregnant women receiving maternal and child care still showed low adherence to prenatal care, late uptake and insufficient HIV testing coverage, which compromised the prevention of vertical transmission of the virus (28). AZT prophylaxis during childbirth was also performed inadequately in several contexts (25). These challenges highlighted the need for new strategies to expand early detection and ensure timely treatment.

In 2002, the “Recommendations for prophylaxis of vertical transmission of HIV and antiretroviral therapy in pregnant women” (*Recomendações para profilaxia da transmissão vertical do HIV e terapia antirretroviral em gestantes*) were published, with guidelines on the use of antiretroviral therapy and case management of the mother-child dyad during childbirth and the postpartum period (29). Also that year, Ordinance No. 2104/2002 established the Nascer-Maternidades Project, which became a landmark in public health policies for reducing vertical transmission of HIV and controlling congenital syphilis, in addition to training multidisciplinary teams and expanding the screening of pregnant women with rapid testing and the provision of prophylactic supplies (AZT, lactation inhibitors and infant formula) (28).

In 2004, results from the Brazilian “Sentinela parturiente” study pointed to regional inequalities in access to testing in the Northeast and North regions of the country (30). In response, the use of rapid HIV detection tests was regulated, especially in hard-to-reach areas and maternity wards (Ordinance No. 34/2005) (31).

In 2007, scientific evidence showed association between maternal HIV viral load of less than 50 copies/mL and reduction in vertical HIV transmission to less than 1% of cases (23,24). This evidence prompted the Ministry of Health to expand the use of combined antiretroviral therapy not only as a method of preventing vertical HIV transmission, but also as treatment for pregnant women living with HIV, considering clinical and immunological criteria (32). In the same year, with the aim of achieving the goal of reducing vertical HIV transmission by 2011, the Operational Plan for the Reduction of Vertical Transmission of HIV and Syphilis (*Plano Operacional para Redução da Transmissão Vertical do HIV e da Sífilis no Brasil*) established tiered targets among the three spheres of public health service management (33).

Despite these initiatives, access to prenatal care and child delivery was still inadequate, and the national program proved to be incipient in terms of health service administration and evaluation (25). In order to improve maternal and child care, strengthen humanized care during pregnancy, childbirth and postpartum, and expand the availability of rapid HIV and syphilis testing in prenatal care, the Rede Cegonha network was established in 2011 (Ordinance No. 1459) (34-36).

In 2013, the WHO updated its HIV treatment guidelines and recommended treatment as prevention for all people living with HIV. Among the measures recommended, initiation of antiretroviral therapy for pregnant and lactating women, regardless of clinical and immunological criteria, to be continued throughout life (option B+) was emphasized (37). In the same year, Brazil stood out internationally by adopting treatment as prevention and expanding universal treatment coverage in the SUS (38). This initiative increased the number of people living with HIV on antiretroviral therapy and resulted in viral suppression in 95% of those in treatment (39). The incorporation of option B+ and pre-treatment genotyping during pregnancy, as well as the expansion of antiretroviral therapy in the postpartum period, increased the likelihood of viral suppression in the first therapeutic regimen (40).

In 2014, the Ministry of Health promoted changes in chemoprophylaxis for exposed newborns and in surveillance strategies for vertical HIV transmission. Considering the findings of the “PACTG 1043” study, it began recommending dual chemoprophylaxis (AZT and nevirapine) for newborns whose mothers did not use antiretrovirals during prenatal care or presented a high HIV viral load (>1,000 copies/mL) in the last trimester of pregnancy. For children whose mothers used antiretroviral therapy during pregnancy, the duration of AZT administration was reduced from 6 to 4 weeks (41). In the same year, the “Protocol for investigating vertical transmission” (*Protocolo de investigação de transmissão vertical*) was launched, which established guidelines for the creation of municipal, state and regional committees responsible for investigating cases and mapping failures in the care pathway (42).

In 2017, Brazil incorporated integrase inhibitors into therapeutic recommendations for pregnant women living with HIV, due to the greater effectiveness of these medications in rapid viral suppression (43). In the same year, aligned with the WHO/PAHO guideline, Brazil launched the subnational certification strategy for the elimination of vertical HIV transmission to recognize progress in municipalities with 100,000 inhabitants or more and promote equity in the response to the epidemic (44).

In 2021, the clinical protocol and therapeutic guidelines for children living with HIV were revised, this time expanding the use of integrase inhibitors for prophylaxis of children exposed to the virus (45,43). The second edition of the national certification guide incorporated the good practice seals (gold, silver and bronze) established by WHO/PAHO, to encourage states and municipalities to achieve indicators close to the elimination criteria (46). By 2024, 79% of eligible municipalities (258/326) and 10 of the 27 federative units had received one of the seals of subnational certification, with a greater concentration in the South and Southeast regions of the country (47,48).

The National Pact for the Elimination of Vertical Transmission of HIV, Syphilis, Hepatitis B and Chagas Disease – aligned with the international agendas of the “Plan of Action Expands the EMTCT Initiative” and the sustainable development goals – was included in the 2024 Healthy Brazil Program (*Programa Brasil Saudável*) to strengthen prevention of diseases that primarily affect vulnerable populations (Figure 2) (49). In 2025, Brazil achieved the global targets and received to WHO/PAHO validation for the elimination of vertical transmission of HIV, consolidating relevant programmatic and institutional advances in maternal and child health (50).

Progressive moves forward in indicators related to prenatal care and treatment of pregnant women living with HIV in Brazil have been demonstrated in studies (51,19). Between 2013 and 2019, prenatal coverage increased from 97% to 98%, the proportion of pregnant women with 6 or more prenatal consultations increased from 84% to 87%, and HIV testing increased from 79% to 99% (51). Use of antiretrovirals during pregnancy evolved from 92% to 94% between 2011 and 2021 (19) (Table 2).

**Table 2:** Characteristics of the studies included in the research and main findings on prenatal care, antiretroviral therapy during pregnancy and gaps in the prevention of vertical transmission of the human immunodeficiency virus (HIV). Brazil, 2019-2025

Author and year Coverage Type of study	Objective	Main results
Rocha et al., 2025 Brazil Cross-sectional study	To analyze indicators related to prenatal care using information from the 2013 and 2019 editions of the National Health Survey.	Between 2013 and 2019, prenatal care coverage remained high and stable (>95.0%), HIV testing increased by about 20.0%, reaching almost 100% (99.3%), while early initiation of prenatal care, the minimum number of consultations, and referral for delivery showed little progress, despite the high coverage of these indicators (>80.0%).
Lima et al., 2025 Brazil Retrospective cohort	To assessing the use of antiretroviral drugs in pregnant women living with HIV in Brazil: a retrospective cohort, 2014-2019.	Among pregnant women, 46.7% had non-White skin color, 27.7% had between 8 and 11 years of schooling, and the median age observed was 27 years. The majority were concentrated in the Southeast (34.6%) and South (30.6%) regions. The North (9.7%) and Midwest (6.8%) regions presented lower proportions.
Ferreira et al., 2025 Brazil National survey	To investigate prevalence and factors associated with adherence to measures to prevent perinatal HIV transmission.	Only 21.0% of the maternity wards evaluated followed all recommendations to prevent vertical transmission of HIV. Locations with greater social vulnerability and fewer monthly births showed lower adherence to prevention measures (OR_a_ 1.2; 95%CI_b_ 0.7; 2.0).
Domingues et al., 2025 Rio de Janeiro Descriptive study	To analyze gestational and maternal outcomes in women with living HIV and evaluate indicators for the prevention of vertical transmission of HIV in Rio de Janeiro.	Between 2021 and 2023, prenatal care coverage was 93.7%, but less than 80.0% had early initiation of care or six consultations. HIV testing reached 79.7%, with 97.2% in public services and 20.6% in the private sector. Use of antiretroviral therapy was recorded on 40.0% of the records, and viral load on 26.6%.
De Lannoy et al., 2024 Border region Ecological study	To analyze the profile and trend of vertical HIV transmission in the Brazilian border region.	Prenatal care coverage was 92.8%, and 69.6% used antiretroviral therapy. Data on delivery, postpartum prophylaxis, and gestational outcome were missing in 38.0% of the records. Among newborns, 51.3% had no records on birth prophylaxis or use of infant formula.
Miranda et al., 2023 Brazil Descriptive study	To assess trends in vertical transmission in Brazil over the last decade (2011-2021).	Between 2011 and 2021, 85,470 pregnant women and 3,781 children were diagnosed with HIV. The incidence rate among pregnant women was 2.5 per 1,000 live births. Incidence rate among children fell from 0.18 to 0.04. Antiretroviral therapy coverage among pregnant women living with HIV remained high, between 92.3% and 94.3%.
Miranda et al., 2023 Brazil Experience report	To describe the subnational certification process for elimination of vertical transmission of HIV and/or syphilis, identifying challenges, barriers and opportunities.	By 2022, 43 municipalities with more than 100,000 inhabitants had been subnationally certified for HIV, syphilis and/or hepatitis B, totaling 24.6 million people, especially in the Southeast and South regions. Despite integration between surveillance and care, shortcomings were revealed in the implementation of electronic medical records and in the recording of prenatal testing.
Aguiar et al., 2023 Rio Branco, Acre Retrospective cohort	To assess the effect of the quality of prenatal and birth care in a cohort of postpartum women cared for in the city of Rio Branco.	63.2% of the pregnant women were aged 20-34, 85.1% were mixed-race, 49.2% had completed high school, and 80.3% belonged to socioeconomic classes C, D and E. Although prenatal care coverage was high (90.9%), inadequate follow-up more than tripled the risk of vertical transmission of HIV, syphilis and hepatitis B (OR 3.49; 95%CI 1.06; 11.52).
Figueredo et al., 2023 São Luís, Maranhão Prospective cohort	To estimate the rate of vertical transmission of HIV in a university hospital in the city of São Luís and to evaluate factors related to vertical transmission of HIV in the period 2013-2017.	86.9% of the pregnant women were over 20 years old, 92.7% were Black, and 46.9% were employed. Prenatal care was received by 86.3%, and antiretroviral therapy was used by 74.6%. During childbirth, 81.8% of mothers and 92.8% of newborns received HIV prophylaxis. Even so, the rate of vertical transmission of HIV was 7.3%, i.e. 4 times higher than the national target of 2.0%.
Perotta et al., 2023 Curitiba, Paraná Cross-sectional study	To describe the sociodemographic and gestational profile of HIV-positive women in the city of Curitiba-PR, from 2018 to 2020.	58.3% of the pregnant women were between 13 and 30 years old, 74.6% were White, and 24.4% had incomplete elementary education. Prenatal care was received by 93.8%, and 82.4% used antiretroviral therapy. During childbirth, 65.1% of the pregnant women and 71.0% of newborns received HIV prophylaxis. Notifiable Health Conditions Information System data showed limitations for assessing the quality of prenatal care.
Pompeu et al., 2022 Pará Analytical retrospective study	To analyze HIV prevalence and associated factors among pregnant women in Pará state.	From 2010 to 2019, average HIV prevalence among pregnant women was 2.4%, predominating in women aged 21-30 years (58.1%), of mixed race (94.3%) and housewives (71.1%). Despite 85.8% prenatal coverage, there was a low number of adequate prenatal consultations (6 or more) (53.9%), use of antiretroviral therapy during pregnancy (74.1%) and knowledge of serological status (15.7%) at the time of child delivery.
Feitoza et al., 2021 Rio Branco, Acre Cross-sectional study	To investigate vertical transmission of HIV in Rio Branco, Acre, Brazil, and to assess the possibility of its elimination.	HIV infection was associated with age ≥20 years and low schooling levels. Prenatal care coverage was 85.7%, but only 36.4% began in the first trimester. Antiretroviral therapy use was below 90.0%, and neonatal prophylaxis reached 85.7%. Case underreporting was found in the Notifiable Health Conditions Information System.
Trindade et al., 2021 Pará Cross-sectional study	To analyze the epidemiological profile of HIV infection in pregnant women.	59.8% of the pregnant women were between 20 and 29 years old, 89.8% were mixed-race, 50.1% had elementary education and 46.8% were housewives. Prenatal care coverage reached 90.5%, but only 34.5% started in the first trimester. Use of antiretroviral therapy during pregnancy was less than 70.0%. There were sociodemographic and access inequalities related to education (p-value<0.001).
Gouvêa et al., 2020 Rio de Janeiro Retrospective cohort	To assess the prevalence of vertical transmission of HIV and associated factors at the Rio de Janeiro State University Hospital, Brazil, from 2007 to 2018.	Average age was 27.4 years, 61.0% were Black, 50.5% had less than 10 years of schooling and 53.6% were unemployed. Prenatal care (90.5%), early initiation in the first trimester, and use of antiretroviral therapy (93.8%) were below 95.0%. Despite high rates of intrapartum (90.4%) and neonatal (98.9%) prophylaxis, the HIV vertical transmission rate reached 2.7%.
Holzmann et al., 2020 Montes Claros, Minas Gerais Retrospective cohort	To assess the implementation of actions to prevent vertical transmission of HIV in two maternity hospitals in Montes Claros, Minas Gerais.	The average age of the pregnant women was 30.2 years, 97.7% were non-White and 54.8% had low education levels (<8 years of schooling). Prenatal care coverage reached 95.7%, and 58.1% had six or more prenatal consultations. Antiretroviral therapy was used during pregnancy in 87.2% of cases, but 62.5% had a detectable viral load at delivery. Among newborns, 84.1% received prophylaxis within the first 24 hours of life, although 30.4% had inadequate case management.
Leal et al., 2020 Brazil Hospital-based national study	To examine regional inequalities in access to and quality of prenatal and childbirth care in public health services in Brazil and their association with perinatal health.	Childbirths were concentrated in adolescents with low levels of education and lower economic status, especially in the North and Northeast regions (p-value<0.001). Prenatal care coverage was 98.5%, but only 56.5% of pregnant women started prenatal care in the first trimester in the North (46.0%) and Northeast (52.2%). 72.1% of the women had six or more prenatal visits. HIV testing occurred in 52.1% of visits.
Freitas et al., 2019 Brazil Descriptive study	To assess the social determinants of access to HIV and VDRL testing during pregnancy in Brazil – National Program for Improving Access and Quality of Primary Care.	Fewer than six prenatal visits were associated with low human development indices (HDI) (≤0.694), low schooling levels (<8 years), and being income transfer programs beneficiaries. Absence of HIV/syphilis testing was related to low HDIs and greater social inequality (Gini≥0.521).
Freitas et al., 2019 Brazil Descriptive study	To evaluate the factors associated with HIV and syphilis testing not being performed during pregnancy in primary health care in Brazil.	Absence of testing was more frequent among White women aged 15-19 years, with low education and social program beneficiaries (PR 1.43; 95%CI 1.19; 1.72). Fewer than six prenatal visits were associated with low education (<8 years) (PR 1.31; 95%CI 1.19; 1.45; p-value<0.001) and receiving social benefits (PR 0.80; 95%CI 0.72; 0.88; p-value<0.001).
Hofer et al., 2019 Rio de Janeiro Retrospective cohort	To assess association between geographic distance, prenatal care coverage and vertical transmission of HIV in Rio de Janeiro.	Living more than 9.5 km from the place of birth significantly reduced the likelihood of receiving prenatal care (OR 0.35; 95%CI 0.18; 0.68). Absence of prenatal care was more prevalent among infected babies (38.1%) compared to uninfected babies (27.3%).

^a^OR: *odds ratio*; ^b^95%CI: 95% confidence interval. ^c^PR: prevalence ratio.

Despite this progress, 89% of pregnant women started prenatal care in the first trimester and had at least 6 consultations (51), with lower performance in the North (49%) and Northeast (55%) regions (52). The lowest coverage rates were observed among pregnant women with less than 8 years of schooling (prevalence ratio 1.31) and beneficiaries of income transfer programs (prevalence ratio 0.80) (53). Adolescent pregnant women, with low education levels, Black or mixed-race, in situations of socioeconomic vulnerability or living in hard-to-reach areas had lower odds of receiving prenatal care (*odds ratio*, OR 0.35) (54,53). Among pregnant women living with HIV, prenatal coverage was 90%, with variations across municipalities, including those in the border region (92%) (55); São Luís (86%) (56); the city of Rio de Janeiro (94%) (57); Curitiba (94%) (58); and Rio Branco (90%) (59) (Table 2).

HIV and syphilis testing was lower in municipalities that had a low human development index (≤0.694) and greater socioeconomic inequality (expressed by the Gini coefficient≥0.521), with higher odds of not being tested among Black pregnant women with up to 8 years of schooling and low income (p-value<0.001) (60) (Table 2).

Regarding use of antiretroviral therapy during pregnancy, the Drug Logistics Control System (*Sistema de Controle Logístico de Medicamentos*) indicated 94% coverage in 2021 (19), while the Notifiable Health Conditions Information System (*Sistema de Informação de Agravos de Notificação*) 70% (61). Pregnant women using antiretroviral therapy had a median age of 27 years, 47% were non-White, 38% were single and 28% had between 8 and 11 years of schooling. The Southeast region concentrated 35% of pregnant women, while the North recorded 10% (62) (Table 2).

The absence of recommended measures for preventing vertical transmission of HIV in maternity wards was more frequent in municipalities with high social vulnerability indices (OR 2.1) (63), including lack of HIV testing, prophylaxis in pregnant women and newborns, and inhibition of lactation (58,64,65) (Table 2).

Municipalities participating in the subnational certification process demonstrated heterogeneity in the use of electronic medical records and weaknesses in the private health services network (66). Studies have shown underreporting of cases of pregnant women living with HIV and lack of data on the quality of prenatal care and maternal and neonatal prophylaxis (55,57,58,66,67) (Table 2).

## Discussion

This study analyzed the evolution of pediatric AIDS cases in Brazil, Brazilian public policies for the elimination of vertical transmission of HIV, and the challenges they face. Brazil has kept pace with scientific advances in the global response to HIV, progressively incorporating innovations in the fields of prevention, diagnosis and treatment. Implementation of strategic programs has favored improvement of prenatal care, use of rapid diagnostic tests, and administration of antiretroviral drugs during pregnancy and child delivery—these being fundamental factors in positioning the country towards the elimination of this disease. However, regional and social inequalities persist in access to services and quality of care, especially in the North and Northeast regions of the country, in addition to structural barriers that limit an equitable response nationwide. The greater concentration of certified municipalities in the South and Southeast regions reflects this scenario.

Despite the expansion of access to prenatal care and the improvement in the quality of care in primary care and maternity wards (28,51), intersectional inequalities of age, education, race/skin color and territory of residence have hindered adequate access to care (53,54), particularly in the North (49%) and Northeast (55%) regions (52).

HIV and syphilis testing coverage has also reflected regional and population disparities, particularly in municipalities with lower socioeconomic status, considering their human development indices and Gini indices. Black pregnant women with up to eight years of schooling and low income were less likely to undergo prenatal testing. Many pregnant women still arrived at maternity wards without knowledge of their own serological status, compromising the timely initiation of prophylaxis at the time of child delivery (60).

This context has highlighted the need to invest in continuing education for health teams, integration between surveillance and care, and strengthening investigation committees, which are essential for identifying failures and proposing corrective actions in the care pathway. Strategies to include partners in prenatal consultations (68) and providing dual HIV and syphilis testing (69) have shown promise in expanding diagnostic coverage and timely initiation of antiretroviral therapy, but effectiveness depends on complementary actions such as counseling and providing combined prevention methods, including pre- and post-exposure prophylaxis.

Despite the broad coverage of pregnant women living with HIV on antiretroviral therapy in Brazil (19), there are discrepancies in information system records, as well as fragmentation, low completeness and heterogeneity of data (55,57,58,66,67), making it necessary to improve workflows and enhance data quality for building health indicators. In South Africa, database triangulation has been useful in the organization, planning and evaluation of indicators related to maternal and child health (70). The National Health Data Network has been seeking to promote interoperability between SUS systems since 2017 (71), but this process is still incomplete, as it depends on investments in infrastructure and training of multidisciplinary teams.

This narrative review sought to describe the topic addressed, expanding knowledge about public policies and future perspectives. The Starlite strategy standardized search and selection criteria, while the use of robust databases and official documents provided representativeness to the analysis of Brazilian policies for the elimination of vertical transmission of HIV.

The study design had limitations, which made it impossible to attribute direct causality between policies implemented and reduction of AIDS cases in children under 5 years old, especially because it did not include intersectoral interventions. Furthermore, the gaps analyzed were based on retrospective studies, which limited its applicability in 2025.

Analysis of the literature revealed a reduction in pediatric AIDS cases in Brazil over four decades, as well as scientific advances incorporated by the SUS for the prevention of vertical transmission of HIV, which contributed to the country achieving the global targets for the elimination of vertical HIV transmission as a public health problem. Despite this important milestone, historical inequities persist in access to prenatal care, timely diagnosis and treatment, especially in the North and Northeast regions of the country. Subnational certification and consolidation of programs to eliminate social diseases (49) can strengthen responses in vulnerable territories. To this end, qualification of local management and continuing education of health professionals are essential for identifying populations in situations of social vulnerability, offering culturally sensitive care, and combating institutional racism (72). Further recommendations include fostering academic debates and conducting research to ensure future generations the right to be born free of HIV, regardless of their territorial origin, social condition, race/skin color, gender and other social markers of difference.

## Data Availability

This narrative review study was based on open-access official documents and scientific publications. The supporting materials used in the narrative review are available in a public repository (73).
