# Supplementary material for: Analysis of the evolution of public policies and strategies for eliminating vertical transmission of the human immunodeficiency virus: a narrative review, Brazil, 1985-2025
Source: Epidemiol Serv Saude. 2026 Jul 20;35:e20250576. doi: 10.1590/S2237-96222026v35e20250576.en (PMC13686289; doi:10.1590/S2237-96222026v35e20250576.en)
Supplement: Translation

# Translation: Análise da evolução das políticas públicas e estratégias para eliminação da transmissão vertical do vírus da imunodeficiência humana: revisão narrativa, Brasil, 1985-2025

**DOI:** 10.1590/S2237-96222026v35e20250576.pt

**Authors:** Andréa Mônica Brandão Beber, Katia Cristina Bassichetto, Francisca Lidiane Sampaio Freitas, Ximena Pamela Díaz Bermúdez, Elisangela da Silva, Lino Neves da Silveira, Leila Posenato Garcia, Adele Schwartz Benzaken

**Affiliations:** 1 Universidade de Brasília Faculdade de Ciências da Saúde Brasília DF Brasil Universidade de Brasília, Faculdade de Ciências da Saúde, Brasília, DF, Brasil; 2 Santa Casa de Misericórdia de São Paulo Faculdade de Ciências Médicas São Paulo SP Brasil Santa Casa de Misericórdia de São Paulo, Faculdade de Ciências Médicas, São Paulo, SP, Brasil; 3 Universidade de Brasília Programa de Pós-Graduação em Saúde Coletiva Brasília DF Brasil Universidade de Brasília, Programa de Pós-Graduação em Saúde Coletiva, Brasília, DF, Brasil; 4 Universidade do Estado do Amazonas Programa de Pós-Graduação em Medicina Tropical Manaus AM Brasil Universidade do Estado do Amazonas, Programa de Pós-Graduação em Medicina Tropical, Manaus, AM, Brasil; 5 Secretaria de Estado da Saúde do Distrito Federal Brasília Brasília DF Brasil Secretaria de Estado da Saúde do Distrito Federal, Brasília, DF, Brasil; 6 Fundação Jorge Duprat Figueiredo de Segurança e Medicina do Trabalho Escritório Avançado em Santa Catarina Florianópolis SC Brasil Fundação Jorge Duprat Figueiredo de Segurança e Medicina do Trabalho, Escritório Avançado em Santa Catarina, Florianópolis, SC, Brasil; 7 AIDS Healthcare Foundation Los Angeles Los Angeles CA Estados Unidos AIDS Healthcare Foundation, Los Angeles, CA, Estados Unidos

**Keywords:** Política de Saúde, Transmissão Vertical de Doenças Infecciosas, HIV, Saúde Materno-Infantil, Revisão Narrativa

## Abstract

**Objetivo:**

Analisar a evolução das políticas públicas de saúde e das estratégias brasileiras voltadas à eliminação da transmissão vertical do vírus da imunodeficiência humana (HIV), a contribuição dessas políticas na redução dos casos de aids em crianças menores de 5 anos e os desafios para alcançar as metas globais de eliminação até 2030.

**Métodos:**

Revisão narrativa de publicações de 1985 a 2025, elaborada a partir da consulta de documentos oficiais do Ministério da Saúde e artigos científicos.

**Resultados:**

A análise de 45 publicações revelou os avanços do Brasil na resposta à transmissão vertical do HIV, com a oferta gratuita de insumos para prevenção, diagnóstico e tratamento do HIV no Sistema Único de Saúde. A implementação de projetos estratégicos, como Nascer-Maternidades e Rede Cegonha, e o processo de certificação subnacional em municípios prioritários contribuíram para o alcance das metas globais de eliminação da transmissão vertical do HIV pelo país em 2025. Iniquidades sociais como raça/cor da pele e classe social, disparidades regionais no cuidado pré-natal, fragilidades nos sistemas de vigilância e barreiras de acesso aos serviços de saúde ainda são desafios para eliminação equitativa desse agravo.

**Conclusão:**

As políticas públicas adotadas no âmbito da saúde materno-infantil foram fundamentais para que o Brasil alcançasse a certificação internacional pela eliminação da transmissão vertical do HIV, como problema de saúde pública. Intensificar programas de mobilização e de educação continuada, fortalecer a gestão local e assegurar a equidade do cuidado são necessários para alcançar as metas em todo território nacional.

**Aspectos éticos**

Esta pesquisa utilizou bancos de dados de domínio público e anonimizados.

## Introdução

As políticas globais para eliminar a transmissão vertical do vírus da imunodeficiência humana (HIV) visam acabar com novas infecções pediátricas até 2030 (1,2). Nas últimas décadas, a Organização Mundial da Saúde (OMS), a Organização Pan-Americana da Saúde (OPAS) e o Fundo das Nações Unidas para a Infância estabeleceram critérios padronizados para validar a eliminação de doenças de transmissão vertical como problemas de saúde pública (3). 

Esse processo teve início em 2005 com o plano estratégico regional da OPAS para o controle do HIV e de outras infecções sexualmente transmissíveis na América Central, expandido em 2007 para as Américas, a Ásia, o Pacífico e a África. Edições sucessivas do “Guia global de validação da eliminação do HIV, sífilis e hepatite B” consolidaram o compromisso internacional de eliminar esses agravos até 2030 (3,4). Alinhadas aos objetivos do desenvolvimento sustentável, essas iniciativas resultaram na redução global de 62% das infecções pediátricas por HIV, passando de 300 mil para 120 mil entre 2010 e 2023 (5). Até 2024, 19 países atingiram alguma das metas de certificação, conforme diferentes critérios de validação (Tabela Suplementar 1) (6,7).

No Brasil, desde 1990, políticas públicas de saúde visam reduzir os casos de aids pediátrica. Em 2017, o país adotou estratégias para atingir a meta internacional de eliminar a transmissão vertical do HIV, visando reduzir essa taxa para menos de 2% e alcançar cobertura pré-natal, testagem para HIV e uso de terapia antirretroviral maior ou igual a 95%, por dois anos consecutivos (8). Entre 2012 e 2023, a taxa de detecção de aids em crianças menores de 5 anos diminuiu cerca de 56%, passando de 3,4 casos para 1,2 caso por 100 mil habitantes (9). 

Apesar desses avanços, persistem barreiras sociorregionais no acesso equitativo ao diagnóstico precoce e ao tratamento durante a gestação. Em 2023, a região Sudeste registrou 31,4% dos casos de gestantes vivendo com HIV, com taxa de detecção de 5,3 casos por 1 mil nascidos vivos, valor superior à média nacional (3,3/1 mil nascidos vivos). No mesmo ano, as maiores taxas de aids em crianças menores de 5 anos concentraram-se nas regiões Norte e Nordeste, com destaque para Roraima (8,2/100 mil habitantes), Pará (2,9/100 mil habitantes) e Sergipe (2,4/100 mil habitantes) (9).

Pesquisas analisaram o impacto de políticas públicas sobre indicadores do HIV no Brasil (10,11), mas há escassez de análises que organizem, de forma histórica e cronológica, a resposta nacional à transmissão vertical do HIV e sua contribuição para a redução dos casos de aids em crianças. Compreender essa trajetória pode fortalecer a adesão às metas pactuadas, aprimorar estratégias regionais e apoiar gestores e formuladores de políticas na identificação de lacunas em contextos de desigualdades, além divulgar boas práticas para outros país e servir de referência para aqueles que buscam eliminar doenças de TV.

Este estudo visou analisar a evolução das políticas públicas de saúde e das estratégias brasileiras voltadas à eliminação da transmissão vertical do HIV, a contribuição dessas políticas na redução dos casos de aids em crianças menores de 5 anos e os desafios para o alcance das metas globais de eliminação até 2030. 

## Métodos

### Delineamento

Trata-se de revisão narrativa da literatura (12,13) desenvolvida com base na análise de documentos técnicos sobre políticas públicas brasileiras voltadas à prevenção da transmissão vertical do HIV, descrição da evolução dos casos de aids em crianças menores de 5 anos e identificação de artigos científicos brasileiros sobre a temática. 

### Estratégia de busca

A estratégia de busca seguiu o acrônimo Starlite (Standards for Reporting Literature Searches) (Tabela 1) (14).

**Tabela 1:** Descrição sumária Standards for Reporting Literature Searches (Starlite) da pesquisa bibliográfica

S	**Estratégia amostral seletiva** Seleção de documentos técnicos do Ministério da Saúde, como manuais, protocolos, portarias e notas técnicas contendo políticas e estratégias voltadas para a redução e eliminação da transmissão vertical do vírus da imunodeficiência humana (HIV). Boletins epidemiológicos com número de casos de aids em menores de 5 anos. Artigos científicos sobre serviços de pré-natal, prevenção da transmissão vertical do HIV e tratamento de gestantes e crianças vivendo com HIV.
T	**Tipos de publicações** Estudos descritivos e avaliativos, revisões de literatura, além de manuais, protocolos, portarias, notas técnicas, legislações e boletins epidemiológicos.
A	Abordagens Pesquisas em bases de dados eletrônicas e sites do Ministério da Saúde.
R	**Intervalo de anos** 1985-2024: inclui dados sobre o número de casos de aids pediátrica, a partir do primeiro caso registrado no Brasil. 1995-2024: abrange o período desde a publicação do primeiro documento nacional sobre políticas programáticas e estratégias de prevenção da transmissão vertical do HIV até 2024. 2019-2025: abrange artigos científicos brasileiros sobre cobertura da assistência pré-natal, testagem para o HIV em gestantes, acesso à terapia antirretroviral durante a gestação e informações da prevenção da transmissão vertical do HIV.
L	Limites Publicações brasileiras, sem restrição de idiomas.
I	**Inclusão e exclusões** Inclusão: documentos técnicos sobre as políticas brasileiras de prevenção da transmissão vertical do HIV e dados epidemiológicos de aids em crianças menores de 5 anos. Exclusão: artigos que não abordavam o tema ou não continham informações relevantes sobre lacunas na prevenção da transmissão vertical.
T	**Termos de busca** Documentos técnicos oficiais e dados sobre aids pediátrica: títulos e ano de publicação. Artigos científicos: Descritores em Ciências da Saúde/Medical Subject Headings (DeCS/MeSH) e operadores booleanos: (((Infectious Disease Transmission, Vertical) OR (Mother-to-Child Transmission OR Vertical Transmission)) AND (HIV)) AND (Brazil). (health coverage) AND (prenatal) AND (brazil) AND (HIV).
E	**Fonte eletrônica de dados** Documentos técnicos oficiais do Ministério da Saúde: Biblioteca Virtual em Saúde e site do Ministério da Saúde (repositórios de conteúdo, vigilância epidemiológica e sistemas de informação), incluindo páginas do antigo Departamento de Doenças Crônicas e Infecções Sexualmente Transmissíveis e do atual Departamento de HIV, Aids, Tuberculose, Hepatites Virais e Infecções Sexualmente Transmissíveis. Dados de casos de aids em menores de 5 anos: boletins epidemiológicos disponíveis no website do Ministério da Saúde, incluindo as mesmas páginas já listadas. Artigos científicos revisados por pares: base de dados PubMed, Scientific Electronic Library Online e Biblioteca Virtual em Saúde/Literatura Latino-Americana e do Caribe em Ciências da Saúde.

### Processo de coleta e seleção

Incluíram-se dois conjuntos de publicações: (i) manuais, protocolos, portarias e notas técnicas, publicados entre 1995 – ano de lançamento do primeiro guia nacional sobre prevenção da transmissão vertical do HIV – e 2024, além de boletins epidemiológicos com dados das primeiras notificações de casos de aids em crianças menores de 5 anos no Brasil, em 1985, até 2024; e (ii) estudos brasileiros sobre cobertura da assistência pré-natal, testagem para HIV em gestantes, acesso à terapia antirretroviral na gestação e profilaxia no parto e no recém-nascido.

Os documentos técnicos foram identificados na Biblioteca Virtual em Saúde e no website do Ministério da Saúde (MS). Dos 35 documentos oficiais selecionados, 26 foram relevantes para analisar políticas e estratégias de eliminação da transmissão vertical do HIV. 

A busca por artigos científicos foi realizada no PubMed, no Scientific Electronic Library Online e na Biblioteca Virtual em Saúde/Literatura Latino-Americana e do Caribe em Ciências da Saúde, e abrangeu publicações de 2019 a 2025, com os descritores: Infectious Disease Transmission, Mother-to-Child Transmission, Vertical Transmission, HIV, Health Coverage, Prenatal e Brazil. Dos 149 artigos encontrados, 59 foram excluídos por estarem duplicados ou fora de escopo, 55 após análise de títulos e resumos, e outros 16 após leitura completa. Foram selecionados 19 artigos.

Para organizar os dois conjuntos de publicações mencionados, foi necessário criar planilhas intermediárias com os conteúdos a seguir.

- Para os documentos técnicos: ano, política/estratégia e mudanças nas diretrizes nacionais.
- Para os artigos: autor, ano, abrangência, características sociodemográficas, cobertura de pré-natal, testagem e tratamento para HIV e manejo no parto.

## Resultados

A seleção final totalizou 45 publicações, sendo 26 documentos técnicos e 19 artigos científicos (Figura 1). 

**Figura 1:** Processo de seleção das publicações para a revisão narrativa sobre políticas públicas brasileiras para eliminação da transmissão vertical do vírus da imunodeficiência humana (HIV). Brasil, 2025

Os documentos técnicos foram ordenados cronologicamente e categorizados segundo a evolução epidemiológica dos casos de aids em crianças menores de 5 anos, concomitantemente aos principais marcos programáticos das políticas e estratégias de prevenção adotadas no país (Figura 2). Os artigos científicos abordaram os desafios para alcançar as metas globais de eliminação.

**Figura 2:** Evolução da taxa de detecção de aids em crianças menores de 5 anos (por 100 mil habitantes), intervenções programáticas e estratégias nacionais para eliminação da transmissão vertical do vírus da imunodeficiência humana (TV-HIV). Brasil, 1985-2025

Os resultados da revisão ilustram o histórico da epidemia e mostram que a aids pediátrica no Brasil foi registrada pela primeira vez em 1985, com três casos notificados no estado de São Paulo (15). Nos anos seguintes, a taxa em menores de 5 anos aumentou progressivamente, atingindo 3,7 casos por 100 mil habitantes em 1995, acompanhando a expansão da epidemia entre mulheres (16). 

Entre 1996 e 2002, o tratamento para a aids foi disponibilizado no Sistema Único de Saúde (SUS), mas subsistiam desafios como acesso limitado ao diagnóstico de HIV, baixa cobertura de testagem no pré-natal e adesão insuficiente à profilaxia existente à época (zidovudina/AZT) durante o parto (16). Ao final desse período, a taxa de incidência de aids pediátrica alcançou o pico na série histórica, com 6,4 casos por 100 mil habitantes, correspondendo a 2,9% das pessoas vivendo com HIV do país (17). 

A partir de 2003, observou-se redução progressiva da taxa de detecção de aids em crianças menores de 5 anos, passando de 5,6 para 3,4 por 100 mil habitantes em 2011. Esse resultado possivelmente foi associado à introdução dos testes rápidos no pré-natal e nas maternidades, ao uso da terapia antirretroviral profilática durante a gestação, à educação continuada dirigida para profissionais de saúde e à ampliação do conhecimento da doença pela população (18). 

De 2012 a 2023, ações específicas implementadas, como a oferta universal da terapia antirretroviral, o diagnóstico precoce e a profilaxia materna e infantil estratificada por risco, contribuíram para a redução da taxa de detecção que passou de 3,4 casos para 1,2 caso por 100 mil habitantes e permitiu alcançar a meta de impacto da OMS/OPAS, cenário que se manteve em 2025 (19). 

Em todos os períodos analisados, para subsidiar a tomada de decisão por gestores e profissionais de saúde, o MS desenvolveu ferramentas complementares de vigilância e monitoramento dos casos de infecção pelo HIV e óbitos por aids em gestantes e crianças, acessíveis aos estados, aos municípios e ao Distrito Federal (Figura 3).

**Figura 3:** Ferramentas de monitoramento de gestantes vivendo com vírus da imunodeficiência humana (HIV), crianças expostas e infectadas, segundo o ano da primeira publicação. Brasil, 2025

As estratégias para redução e eliminação da transmissão vertical do HIV iniciaram-se após a publicação do ensaio clínico Protocolo 076 do AIDS Clinical Trial Group (“PACTG 076”) em 1994, nos Estados Unidos. Esse estudo representou um marco inicial no manejo de gestantes vivendo com HIV, demonstrando a eficácia do AZT na redução da transmissão vertical do HIV quando administrado durante a gestação, no parto e ao recém-nascido não amamentado (20). No ano seguinte, o Brasil publicou as primeiras diretrizes nacionais para profissionais de saúde com orientações para a prevenção da transmissão vertical do HIV, reunidas nas “Considerações gerais do binômio: HIV/aids e gravidez” (15). 

As medidas, no entanto, foram implementadas apenas após promulgação da Lei nº 9.313/1996, que instituiu a distribuição gratuita de antirretrovirais no SUS e buscou assegurar acesso universal à terapia antirretroviral para pessoas vivendo com HIV com indicação terapêutica (21). Havia, porém, incertezas sobre os mecanismos de transmissão do HIV à criança e os efeitos em longo prazo do uso do AZT durante a gestação (22).

Entre 1996 e 2001, estudos reconhecidos mundialmente evidenciaram a importância dos fatores virológicos e imunológicos maternos na transmissão vertical do HIV, principalmente nas semanas finais da gestação, durante o parto e no aleitamento (20,23,24). Em 1996, o Brasil passou a oferecer AZT intravenoso na rede pública para profilaxia no parto (25). Em 1997, a Portaria nº 874 do MS reforçou o uso do AZT a partir da 14ª semana gestacional independentemente de fatores clínicos e imunológicos, incorporou a terapia combinada para gestantes vivendo com HIV imunossuprimidas, introduziu os inibidores de protease para gestantes em falha terapêutica e estendeu a profilaxia com AZT ao recém-nascido da quarta para a sexta semana de vida, mantendo a indicação de cesariana eletiva e a contraindicação da amamentação (26). 

No início dos anos 2000, o aumento dos casos de HIV entre mulheres em idade reprodutiva e gestantes evidenciou a necessidade de políticas de prevenção mais robustas. Em resposta, o MS instituiu a notificação compulsória de gestantes vivendo com HIV e de crianças expostas ao vírus, por meio da Portaria nº 993/2000, o que intensificou a vigilância do agravo e aprimorou o cuidado direcionado a essas populações (27).

Embora o Brasil oferecesse insumos para diagnóstico e tratamento da aids no SUS, as gestantes atendidas na assistência materno-infantil ainda apresentavam baixa adesão ao pré-natal, captação tardia e cobertura insuficiente de testagem para HIV, o que comprometeu a prevenção da transmissão vertical do vírus (28). A profilaxia com AZT durante o parto também era realizada de maneira inadequada em diversos contextos (25). Esses desafios evidenciaram a necessidade de novas estratégias para ampliar a detecção precoce e assegurar o tratamento oportuno.

Em 2002, publicaram-se as “Recomendações para profilaxia da transmissão vertical do HIV e terapia antirretroviral em gestantes”, com diretrizes sobre o uso da terapia antirretroviral e o manejo do binômio no parto e no puerpério (29). Nesse mesmo ano, a Portaria nº 2.104/2002 instituiu o Projeto Nascer-Maternidades, que se tornou um marco nas políticas públicas de saúde para redução da transmissão vertical do HIV e controle da sífilis congênita. A iniciativa capacitou equipes multiprofissionais e ampliou a triagem de gestantes com teste rápido e a oferta de insumos profiláticos (AZT, inibidores de lactação e fórmula láctea) (28). 

Em 2004, resultados do estudo brasileiro “Sentinela parturiente” apontavam desigualdades regionais no acesso à testagem nas regiões Nordeste e Norte do país (30). Como resposta, regulamentou-se o uso do teste rápido para detecção do HIV, especialmente em áreas de difícil acesso e maternidades (Portaria nº 34/2005) (31).

Em 2007, evidências científicas mostraram associação entre a carga viral do HIV materna inferior a 50 cópias/mL e a redução da transmissão vertical do HIV para menos de 1% dos casos (23,24). Tal evidência impulsionou o MS a ampliar o uso da terapia antirretroviral combinada não apenas como método de prevenção da transmissão vertical do HIV, mas também como tratamento para as gestantes vivendo com HIV, considerando critérios clínicos e imunológicos (32). No mesmo ano, com o intuito de alcançar a meta de reduzir a transmissão vertical do HIV até 2011, o Plano Operacional para a Redução da Transmissão Vertical do HIV e da Sífilis estabeleceu metas escalonadas entre as três esferas da gestão pública (33).

Apesar dessas iniciativas, o acesso ao pré-natal e ao parto ainda era inadequado, e o programa nacional mostrou-se incipiente quanto à administração e à avaliação dos serviços de saúde (25). Para qualificar a assistência materno-infantil, fortalecer o atendimento humanizado na gestação, parto e puerpério e ampliar a oferta de teste rápido de HIV e sífilis no pré-natal, foi instituída a Rede Cegonha em 2011 (Portaria nº 1.459) (34-36).

Em 2013, a OMS atualizou as diretrizes terapêuticas do HIV e recomendou o tratamento como prevenção para todas as pessoas vivendo com HIV. Entre as medidas preconizadas, ressaltou-se o início da terapia antirretroviral para gestantes e lactantes, independentemente dos critérios clínicos e imunológicos, com manutenção ao longo da vida (opção B+) (37). No mesmo ano, o Brasil se destacou internacionalmente ao adotar o tratamento como prevenção e ampliar a cobertura do tratamento universal no SUS (38). Essa iniciativa aumentou o número de pessoas vivendo com HIV em terapia antirretroviral e resultou na supressão viral de 95% daquelas em tratamento (39). A incorporação da opção B+ e da genotipagem pré-tratamento durante a gestação, bem como a ampliação da terapia antirretroviral no puerpério aumentaram a chance de supressão viral no primeiro esquema terapêutico (40). 

Em 2014, o MS promoveu mudanças na quimioprofilaxia dos recém-nascidos expostos e nas estratégias de vigilância da transmissão vertical do HIV. Considerando os achados do estudo “PACTG 1043”, passou a recomendar a quimioprofilaxia dupla (AZT e nevirapina) para recém-nascidos cujas mães não utilizaram antirretrovirais no pré-natal ou apresentaram carga viral do HIV elevada (>1 mil cópias/mL) no último trimestre gestacional. Para crianças cujas mães utilizaram terapia antirretroviral na gestação, o tempo de administração de AZT foi reduzido de 6 para 4 semanas (41). No mesmo ano, foi lançado o “Protocolo de investigação de transmissão vertical”, que estabeleceu diretrizes para a criação de comitês municipais, estaduais e regionais responsáveis pela investigação dos casos e mapeamento das falhas na linha de cuidado (42). 

Em 2017, o Brasil incorporou os inibidores da integrase nas recomendações terapêuticas para gestantes vivendo com HIV, devido à maior eficácia desses medicamentos na rápida supressão viral (43). No mesmo ano, alinhado ao guia da OMS/OPAS, o país lançou a estratégia de certificação subnacional da eliminação da transmissão vertical do HIV para reconhecer avanços de municípios com 100 mil habitantes ou mais e promover a equidade na resposta à epidemia (44).

Em 2021, revisou-se o protocolo clínico e diretrizes terapêuticas para crianças vivendo com HIV, dessa vez ampliando o uso dos inibidores da integrase para profilaxia das crianças expostas ao vírus (45,43). A segunda edição do guia nacional de certificação incorporou os selos de boas práticas (ouro, prata e bronze) instituídos pela OMS/OPAS, para estimular a adesão de estados e municípios a alcançarem indicadores próximos aos critérios de eliminação (46). Até 2024, 79% dos municípios elegíveis (258/326) e 10 das 27 unidades federativas haviam recebido algum selo da certificação subnacional, com maior concentração nas regiões Sul e Sudeste do país (47,48).

O Pacto Nacional para a Eliminação da Transmissão Vertical de HIV, Sífilis, Hepatite B e Doença de Chagas – alinhado às agendas internacionais do Plan of Action Expands the EMTCT Initiative e aos objetivos do desenvolvimento sustentável – foi incluído no Programa Brasil Saudável, de 2024, para fortalecer a prevenção de doenças que afetam prioritariamente populações vulnerabilizadas (Figura 2) (49). Em 2025, o Brasil atingiu as metas globais e recebeu a certificação internacional da OMS/OPAS pela eliminação da transmissão vertical do HIV, consolidando os avanços programáticos e institucionais relevantes no âmbito da saúde materno-infantil (50).

Avanços progressivos nos indicadores referentes ao cuidado pré-natal e ao tratamento de gestantes vivendo com HIV no Brasil foram demonstrados em estudos (51,19). Entre 2013 e 2019, a cobertura pré-natal passou de 97% para 98%, a proporção de gestantes com 6 ou mais consultas aumentou de 84% para 87%, e a testagem para HIV cresceu de 79% para 99% (51). O uso de antirretrovirais durante a gestação evoluiu de 92% para 94% entre 2011 e 2021 (19) (Tabela 2).

**Tabela 2:** Características dos estudos incluídos na pesquisa e principais achados sobre assistência pré-natal, terapia antirretroviral na gestação e lacunas na prevenção da transmissão vertical do vírus da imunodeficiência humana (HIV). Brasil, 2019-2025

Autor e ano Abrangência Tipo de estudo	Objetivo	Principais resultados
Rocha et al., 2025 Brasil Estudo transversal	Analisar indicadores relacionados ao pré-natal utilizando as informações das edições da Pesquisa Nacional de Saúde de 2013 e 2019.	Entre 2013 e 2019, a cobertura de pré-natal manteve-se alta e estável (>95,0%), a testagem para HIV ampliou em cerca de 20,0% atingindo praticamente a totalidade (99,3%), o início precoce do pré-natal, o número mínimo de consultas e a referência para o parto tiveram poucos avanços, apesar da alta cobertura desses indicadores (>80,0%).
Lima et al., 2025 Brasil Coorte retrospectivo	Avaliar o uso de antirretrovirais em gestantes vivendo com HIV no Brasil: uma coorte retrospectiva, 2014-2019.	Entre as gestantes, 46,7% tinham cor de pele não branca, 27,7% possuíam entre 8 e 11 anos de escolaridade, e a idade mediana observada foi de 27 anos. A maioria se concentrou nas regiões Sudeste (34,6%) e Sul (30,6%). Norte (9,7%) e Centro-Oeste (6,8%) apresentaram menores proporções.
Ferreira et al., 2025 Brasil Inquérito nacional	Investigar a prevalência e os fatores associados à adesão às medidas de prevenção da transmissão perinatal do HIV.	Apenas 21,0% das maternidades avaliadas seguiram todas as recomendações para prevenir a transmissão vertical do HIV. Locais com maior vulnerabilidade social e menos partos mensais apresentaram menor adesão às medidas preventivas (OR_a_ 1,2; IC95%_b_ 0,7; 2,0).
Domingues et al., 2025 Rio de Janeiro Estudo descritivo	Analisar desfechos gestacionais e maternos das mulheres com HIV e avaliar indicadores para prevenção da transmissão vertical do HIV no Rio de Janeiro.	Entre 2021 e 2023, a cobertura de pré-natal foi de 93,7%, mas menos de 80,0% tiveram início precoce do acompanhamento ou seis consultas. O teste de HIV alcançou 79,7%, sendo 97,2% no serviço público e 20,6% no privado. O uso da terapia antirretroviral foi registrado em 40,0% das cadernetas e da carga viral em 26,6%.
De Lannoy et al., 2024 Faixa de fronteira Estudo ecológico	Analisar o perfil e a tendência da transmissão vertical do HIV na faixa de fronteira brasileira.	A cobertura pré-natal foi de 92,8%, e 69,6% fizeram uso de terapia antirretroviral. Em 38,0% dos registros, faltavam dados sobre o parto, a profilaxia da puérpera e o desfecho gestacional. Entre os recém-nascidos, 51,3% não tinham anotações sobre profilaxia ao nascimento ou uso da fórmula infantil.
Miranda et al., 2023 Brasil Estudo descritivo	Avaliar as tendências da transmissão vertical no Brasil na última década (2011-2021).	Entre 2011 e 2021, houve 85.470 gestantes e 3.781 crianças diagnosticadas com HIV. A taxa de incidência entre as gestantes foi de 2,5 para cada 1 mil nascidos vivos. A taxa de incidência entre as crianças caiu de 0,18 para 0,04. A cobertura da terapia antirretroviral permaneceu alta, entre 92,3% e 94,3%.
Miranda et al., 2023 Brasil Relato de experiência	Descrever o processo de certificação subnacional da eliminação da transmissão vertical de HIV e/ou sífilis, identificando desafios, barreiras e oportunidades.	Até 2022, 43 municípios com mais de 100 mil habitantes foram certificados subnacionalmente para o HIV, sífilis e/ou hepatite B, totalizando 24,6 milhões de pessoas, especialmente nas regiões Sudeste e Sul. Apesar da integração entre vigilância e assistência, revelaram-se falhas na implementação do prontuário eletrônico e no registro de testagens no pré-natal.
Aguiar et al., 2023 Rio Branco, Acre Coorte retrospectiva	Avaliar o efeito da qualidade dos cuidados pré-natais e nascimento em uma coorte de puérperas atendidas na cidade de Rio Branco.	Entre as gestantes, 63,2% tinham 20-34 anos, 85,1% eram pardas, 49,2% tinham ensino médio e 80,3% pertenciam às classes C, D e E. Embora a cobertura pré-natal tenha sido elevada (90,9%), a inadequação do acompanhamento aumentou em mais de três vezes o risco de transmissão vertical de HIV, sífilis e hepatite B (OR 3,49; IC95% 1,06; 11,52).
Figueredo et al., 2023 São Luis, Maranhão Coorte prospectiva	Estimar a taxa de transmissão vertical do HIV em um hospital universitário de referência em São Luís e avaliar os fatores relacionados à transmissão vertical do HIV no período 2013-2017.	Entre as gestantes, 86,9% tinham mais de 20 anos, 92,7% eram negras e 46,9% estavam empregadas. O pré-natal foi realizado por 86,3% e o uso de terapia antirretroviral por 74,6%. Durante o parto, 81,8% das parturientes e 92,8% dos recém-nascidos receberam profilaxia para HIV. Ainda assim, a taxa de transmissão vertical do HIV ficou em 7,3%, superando em quatro vezes a meta nacional de 2,0%.
Perotta et al., 2023 Curitiba, Paraná Estudo transversal	Descrever o perfil sociodemográfico e gestacional de mulheres HIV positivas da cidade de Curitiba-PR, de 2018 a 2020.	Entre as gestantes, 58,3% tinham entre 13 e 30 anos, 74,6% eram brancas e 24,4% possuíam ensino fundamental incompleto. O pré-natal foi realizado por 93,8% e 82,4% fizeram uso de terapia antirretroviral. Durante o parto, 65,1% das gestantes e 71,0% dos recém-nascidos receberam profilaxia para o HIV. Os dados provenientes do Sistema de Informação de Agravos de Notificação mostraram limitações para avaliar a qualidade do pré-natal.
Pompeu et al., 2022 Pará Estudo analítico, retrospectivo	Analisar a prevalência do HIV e fatores associados em gestantes no Pará.	De 2010 a 2019, a prevalência média de HIV em gestantes foi de 2,4%, predominando em mulheres de 21-30 anos (58,1%), pardas (94,3%) e donas do lar (71,1%). Apesar da cobertura pré-natal de 85,8%, houve baixo número adequado de seis ou mais consultas (53,9%), uso da terapia antirretroviral durante a gestação (74,1%) e do conhecimento do status sorológico (15,7%) no momento do parto.
Feitoza et al., 2021 Rio Branco, Acre Estudo transversal	Verificar a transmissão vertical do HIV em Rio Branco, Acre, Brasil, e avaliar a possibilidade de eliminação.	A infecção pelo HIV esteve relacionada à idade ≥20 anos e à baixa escolaridade. A cobertura pré-natal foi de 85,7%, mas apenas 36,4% começaram no primeiro trimestre. O uso de terapia antirretroviral ficou abaixo de 90,0%, e a profilaxia neonatal atingiu 85,7%. Encontrou-se subnotificação dos casos no Sistema de Informação de Agravos de Notificação.
Trindade et al., 2021 Pará Estudo transversal	Analisar o perfil epidemiológico da infecção pelo HIV em gestantes.	Entre as gestantes, 59,8% tinham entre 20 e 29 anos, 89,8% eram pardas, 50,1% tinham ensino fundamental e 46,8% eram donas de casa. O pré-natal atingiu cobertura de 90,5%, porém só 34,5% iniciaram no primeiro trimestre. O uso da terapia antirretroviral na gestação foi inferior a 70,0%. Houve desigualdades sociodemográficas e de acesso relacionadas à escolaridade (p-valor<0,001).
Gouvêa et al., 2020 Rio de Janeiro Coorte retrospectivo	Avaliar a prevalência transmissão vertical do HIV e fatores associados no Hospital Universitário da Universidade Estadual do Rio de Janeiro, Brasil, de 2007 a 2018.	A média de idade foi 27,4 anos, 61,0% eram negras, 50,5% tinham menos de 10 anos de estudo e 53,6% não tinham remuneração. A realização do pré-natal (90,5%), o início precoce no primeiro trimestre e o uso da terapia antirretroviral (93,8%) ficaram abaixo de 95,0%. Apesar da alta profilaxia intraparto (90,4%) e neonatal (98,9%), a taxa de transmissão vertical do HIV atingiu 2,7%.
Holzmann et al., 2020 Montes Claros, Minas Gerais Coorte retrospectivo	Avaliar a implementação das ações de prevenção da transmissão vertical do HIV em duas maternidades de Montes Claros, Minas Gerais.	A média de idade das gestantes foi 30,2 anos, 97,7% não brancas e 54,8% baixa escolaridade (<8 anos de estudo). A cobertura pré-natal atingiu 95,7% e 58,1% realizaram seis ou mais consultas. O uso de terapia antirretroviral durante a gestação ocorreu em 87,2%, mas 62,5% tinham carga viral detectável no parto. Entre os recém-nascidos, 84,1% receberam profilaxia nas primeiras 24 horas de vida, embora 30,4% tenham tido manejo inadequado.
Leal et al., 2020 Brasil Estudo de base hospitalar nacional	Verificar desigualdades regionais no acesso e na qualidade da atenção ao pré-natal e ao parto nos serviços públicos de saúde no Brasil e a sua associação com a saúde perinatal.	Os partos concentraram-se entre adolescentes, com baixa escolaridade e menor nível econômico, sobretudo no Norte e no Nordeste (p-valor<0,001). A cobertura pré-natal foi de 98,5%, mas apenas 56,5% das gestantes iniciaram no primeiro trimestre no Norte (46,0%) e no Nordeste (52,2%). Seis ou mais consultas foram realizadas por 72,1% das mulheres. A testagem para HIV ocorreu em 52,1% das consultas.
Freitas et al., 2019 Brasil Estudo descritivo	Avaliar os determinantes sociais do acesso aos testes de HIV e VDRL durante a gestação no Brasil – Programa Nacional de Melhoria do Acesso e da Qualidade da Atenção Básica.	Menos de seis consultas de pré-natal foi associado ao baixo índice de desenvolvimento humano – IDH (≤0,694), à baixa escolaridade (<8 anos) e à participação em programas de transferência de renda. A ausência de testagem para HIV/sífilis relacionou-se ao baixo IDH e à maior desigualdade social (Gini≥0,521).
Freitas et al., 2019 Brasil Estudo descritivo	Avaliar os fatores associados à não realização de testagem para HIV e sífilis durante a gestação na atenção primária à saúde no Brasil.	A ausência de testagem foi mais frequente entre mulheres brancas de 15-19 anos, com baixa escolaridade e beneficiárias de programas sociais (RP_c_ 1,43; IC95% 1,19; 1,72). Menos de seis consultas de pré-natal associou-se à baixa escolaridade (<8 anos) (RP 1,31; IC95% 1,19; 1,45; p-valor<0,001) e ao recebimento de benefícios sociais (RP 0,80; IC95% 0,72; 0,88; p-valor<0,001).
Hofer et al., 2019 Rio de Janeiro Coorte retrospectivo	Avaliar a associação entre distância geográfica, cobertura de pré-natal e transmissão vertical do HIV no Rio de Janeiro.	Morar a mais de 9,5 km do local do parto reduziu significativamente a chance de realizar o pré-natal (OR 0,35; IC95% 0,18; 0,68). A ausência de pré-natal foi mais prevalente entre bebês infectados (38,1%) em comparação aos não infectados (27,3%).

^a^OR: razão de chances (*odds ratio*); ^b^IC95%: intervalo de confiança de 95%. ^c^RP: razão de prevalência.

Apesar desse progresso, 89% das gestantes iniciaram o pré-natal no primeiro trimestre e realizaram o mínimo de 6 consultas (51), com menor desempenho nas regiões Norte (49%) e Nordeste (55%) (52). As menores coberturas foram observadas entre gestantes com menos de 8 anos de estudo (razão de prevalência 1,31) e beneficiárias de programas de transferência de renda (razão de prevalência 0,80) (53). Gestantes adolescentes, com baixa escolaridade, negras ou pardas, em situação de vulnerabilidade socioeconômica ou residentes em áreas de difícil acesso tiveram menor chance de realização do pré-natal (razão de chances – *odds ratio*, OR 0,35) (54,53). Entre as gestantes vivendo com HIV, a cobertura pré-natal foi 90%, com variações entre municípios, incluindo a faixa de fronteira (92%) (55), São Luís (86%) (56), Rio de Janeiro (94%) (57), Curitiba (94%) (58) e Rio Branco (90%) (59) (Tabela 2).

A testagem para HIV e sífilis foi inferior em municípios que apresentaram baixo índice de desenvolvimento humano (≤0,694) e maior desigualdade socioeconômica (expressa pelo coeficiente de Gini≥0,521), com maior chance de não testagem entre gestantes negras, com até 8 anos de escolaridade e baixa renda

(p-valor<0,001) (60) (Tabela 2).

Com relação ao uso de terapia antirretroviral durante a gestação, o Sistema de Controle Logístico de Medicamentos indicou cobertura de 94% em 2021 (19), enquanto o Sistema de Informação de Agravos de Notificação registrou 70% (61). Gestantes vivendo com HIV em uso de terapia antirretroviral tinham idade mediana de 27 anos, 47% eram não brancas, 38% solteiras e 28% tinham entre 8 e 11 anos de escolaridade. A região Sudeste concentrou 35% das gestantes, enquanto o Norte registrou 10% (62) (Tabela 2).

A ausência das medidas recomendadas para prevenção da transmissão vertical do HIV nas maternidades teve maior frequência em municípios com índices elevados de vulnerabilidade social (OR 2,1) (63), incluindo falta de testagem para HIV, profilaxia em gestantes e recém-nascidos e inibição da lactação (58,64,65) (Tabela 2).

Municípios partícipes do processo de certificação subnacional demonstraram heterogeneidade no uso do prontuário eletrônico e fragilidades na rede privada de serviços de saúde (66). Estudos mostraram subnotificação de casos de gestantes vivendo com HIV e ausência de dados sobre a qualidade do pré-natal e da profilaxia materna e neonatal (55,57,58,66,67) (Tabela 2).

## Discussão

Este estudo analisou a evolução dos casos de aids pediátrica no Brasil, as políticas públicas brasileiras para eliminação da transmissão vertical do HIV e seus desafios. O país acompanhou avanços científicos na resposta global ao HIV, incorporando progressivamente inovações nos campos de prevenção, diagnóstico e tratamento. A implementação de programas estratégicos favoreceu a qualificação do cuidado pré-natal, o uso de testes rápidos para diagnóstico e a administração de antirretrovirais durante a gestação e o parto, fatores fundamentais para posicionar o país rumo à eliminação desse agravo. Persistem, porém, desigualdades regionais e sociais no acesso aos serviços e na qualidade do cuidado, especialmente nas regiões Norte e Nordeste, além de barreiras estruturais que limitam a resposta equitativa em âmbito nacional. A maior concentração de municípios certificados nas regiões Sul e Sudeste reflete esse cenário.

Apesar da ampliação do acesso ao pré-natal e do aprimoramento da qualidade da assistência na atenção básica e maternidades (28,51), desigualdades interseccionais de idade, escolaridade, raça/cor da pele e território de moradia dificultaram o acesso adequado ao cuidado (53,54) com destaque para regiões Norte (49%) e Nordeste (55%) (52). 

A cobertura dos testes de HIV e sífilis também refletiu disparidades regionais e populacionais, principalmente em municípios em pior posição socioeconômica considerando o índice de desenvolvimento humano e de Gini. Gestantes negras com até oito anos de escolaridade e baixa renda apresentaram menor probabilidade de realizar exames no pré-natal. Muitas gestantes ainda chegavam às maternidades sem conhecimento do próprio estado sorológico, comprometendo o início oportuno da profilaxia no momento do parto (60).

Esse contexto evidencia a necessidade de investir na formação continuada das equipes de saúde, na integração entre vigilância e assistência e no fortalecimento dos comitês de investigação, essenciais para identificar falhas e propor ações corretivas na linha do cuidado. Estratégias para incluir parcerias nas consultas (68) e a oferta de teste duplo de HIV e sífilis (69) têm se mostrado promissoras para ampliar a cobertura diagnóstica e o início oportuno da terapia antirretroviral, mas a efetividade depende de ações complementares como aconselhamento e a oferta de métodos de prevenção combinada, incluindo profilaxias pré e pós-exposição. 

Apesar da ampla cobertura de gestantes vivendo com HIV em terapia antirretroviral no Brasil (19), existem divergências nos registros dos sistemas de informação, bem como fragmentação, baixa completude e heterogeneidade dos dados (55,57,58,66,67), sendo necessário aprimorar os fluxos e melhorar a qualidade dos dados na construção de indicadores de saúde. Na África do Sul, a triangulação de bases tem sido útil na organização, no planejamento e na avaliação de indicadores relacionados à saúde materno-infantil (70). A Rede Nacional de Dados em Saúde busca promover a interoperabilidade entre os sistemas do SUS desde 2017 (71), mas esse processo ainda está inconcluso, pois depende de investimentos em infraestrutura e capacitação de equipes multiprofissionais.

Esta revisão narrativa buscou descrever o tema abordado, ampliando o conhecimento sobre políticas públicas e perspectivas futuras. A estratégia Starlite padronizou critérios de busca e seleção, enquanto o uso de bases robustas e documentos oficiais conferiu representatividade à análise das políticas brasileiras para eliminação da transmissão vertical do HIV. 

Este delineamento apresentou limitações, as quais impossibilitaram atribuir causalidade direta entre políticas implementadas e redução dos casos de aids em crianças menores de 5 anos, especialmente por não contemplar intervenções intersetoriais. Além disso, as lacunas analisadas basearam-se em estudos retrospectivos, o que restringiu a aplicabilidade ainda em 2025.

A análise da literatura evidenciou a redução dos casos de aids pediátrica no Brasil ao longo de quatro décadas, bem como os avanços científicos incorporados pelo SUS para a prevenção da transmissão vertical do HIV, os quais contribuíram para que o país alcançasse as metas globais de eliminação da transmissão vertical do HIV como problema de saúde pública. Apesar desse importante marco, perpetuam-se iniquidades históricas no acesso ao pré-natal, diagnóstico oportuno e tratamento, sobretudo nas regiões Norte e Nordeste. A certificação subnacional e a consolidação de programas para eliminar doenças sociais (49) podem fortalecer respostas nos territórios vulnerabilizados. Para isso, a qualificação da gestão local e a educação permanente dos profissionais de saúde são essenciais para identificar populações em situação de vulnerabilidade social, oferecer cuidados culturalmente sensíveis e combater o racismo institucional (72). Também, recomenda-se fomentar debates acadêmicos e realizar pesquisas para assegurar às futuras gerações o direito de nascerem livres do HIV, independentemente de sua origem territorial, condição social, raça/cor da pele, gênero entre outros marcadores sociais da diferença.

## Data Availability

Este estudo de revisão narrativa foi baseado em documentos oficiais de livre acesso e publicações científicas. Os materiais de apoio utilizados na revisão narrativa estão disponíveis em repositório público no Zenodo: (73).
